# AS03 stresses out macrophages: Commentary on ‘Activation of the endoplasmic reticulum stress sensor IRE1α by the vaccine adjuvant AS03 contributes to its immunostimulatory properties’

**DOI:** 10.1038/s41541-018-0070-8

**Published:** 2018-06-28

**Authors:** Mark T. Orr, Christopher B. Fox

**Affiliations:** 1 Infectious Disease Research Institute, Seattle, WA 98102 USA; 2 Department of Global Health, University of Washington, Seattle, WA 98185 USA

Modern vaccine development has placed a large focus on the development of rationally designed immunogens for difficult-to-prevent diseases including HIV, universal influenza, and RSV. Adjuvants are often required to program the desired immune response to these otherwise immunologically inert antigens. Next to aluminum salts, oil-in-water (o/w) emulsions represent the most widely employed class of vaccine adjuvant formulations in licensed human vaccines, with the number of doses administered (primarily as a component of influenza vaccines) now counted in the hundreds of millions.^1^ With regard to influenza vaccines, the most important adjuvant properties of o/w emulsions include antigen dose sparing (a key attribute for pandemic preparedness), broadening of antibody responses to protect against heterologous strains, and enhancement of protective immune responses in susceptible populations including young children and the elderly. Several o/w emulsion adjuvants are included in licensed human vaccines (e.g., MF59 in Fluad® and AS03 in Pandemrix®) or are in clinical development.

Commensurate with the extensive usage of o/w emulsions as adjuvants, multiple studies in recent years have sought to elucidate the innate immune mechanisms of action responsible for their immunostimulatory properties. O/w emulsions rapidly traffic to the local draining lymph node where they are captured by subscapsular macrophages that line the draining lymph node and activate CD11b^+^ cells including macrophages, monocytes, and neutrophils.^2–4^ These cells, along with dendritic cells, then capture the co-injected vaccine antigen and present it to naive T cells. Additionally, o/w emulsions elicit production of chemokine and cytokine gradients that recruit inflammatory cells to the injection site and to the draining lymph node. Similar rapid increases in monocytes and chemokines have been found in the peripheral of healthy humans receiving o/w adjuvanted influenza vaccines.^5,6^ The production of the danger-associated molecular pattern (DAMP) molecule extracellular ATP, activation of the ASC-dependent inflammasome, and one or more MyD88-dependent receptors (e.g., IL-1R, IL-18R, or TLRs) are critical for the generation of this pro-inflammatory environment and subsequent adaptive immune response.^7–10^ Importantly, the precise formulation of these o/w adjuvants is necessary for their adjuvant activity.^2,3^

Despite the increasing understanding regarding the biological mechanisms of action relevant for o/w emulsion adjuvant activity, extensive knowledge gaps in molecular mechanisms remain. The study by Givord et al. published in the current issue of *npj Vaccines*, uses transcriptional profiling methods to identify the endoplasmic reticulum (ER) stress sensor kinase IRE1α as a key regulator of cytokine production in myeloid cells stimulated by AS03 in vitro and in vivo.^11^ They go on to determine that this pathway subsequently impacts the magnitude of the resultant T follicular helper (TFH) cell response and antibody avidity. The activation of IRE1α was associated with changes in cellular lipid content and ER morphology induced by AS03. While it was previously known that uptake of o/w emulsion adjuvants causes changes in cellular lipid metabolism,^12^ the present work expanded on the relevant downstream signaling mediators of the unfolded protein response (UPR), elucidating the hitherto unappreciated role of the IRE1α/TRAF2/ASK1/JNK pathway in the adjuvant activity of a leading o/w emulsion adjuvant. Using a selective gene deletion system, the authors demonstrate that IRE1α expression by myeloid cells contributes to the in vivo elicitation of TFH cells by the AS03 adjuvant, likely by augmenting IL-6 production from the innate immune response. This newly defined mechanism of AS03’s action is consistent with a growing consensus that particulate adjuvants such as o/w emulsions induce DAMPs, resulting in activation of inflammatory pathways.^8–10^ There is also an increasing body of evidence illustrating the role of the UPR in many aspects of innate and adaptive immunity.^13,14^ The transcription factors XBP-1 and ATF6B, key mediators of the UPR pathway, were identified as major components of the transcriptional response to seasonal influenza vaccine.^15^

While identification of the role of IRE1α represents another piece of the puzzle for o/w emulsion mechanisms of activity, much work remains to be done. The most important question, of course, is how will adjuvant scientists and vaccine developers leverage this new finding to develop safer or more effective vaccine adjuvants? More specifically, it will be important to determine the contributions of each of the components of AS03 (squalene, α-tocopherol, and polysorbate 80) as well as the relevance of the study’s conclusions for different adjuvant compositions (o/w emulsions or other formulations). Likewise, additional studies will be needed to determine the role of other UPR pathways that are not dependent on IRE1α, and whether these mechanisms of adjuvanticity identified in model species are representative of human biology.^15^ Such knowledge will clarify and augment the current knowledge base regarding mechanisms of action of o/w emulsion adjuvants, establish the translational impact of the current findings, and help inform next-generation adjuvant design.
